# Cartilage preservation by inhibition of Janus kinase 3 in two rodent models of rheumatoid arthritis

**DOI:** 10.1186/ar2365

**Published:** 2008-01-30

**Authors:** Anthony J Milici, Elizabeth M Kudlacz, Laurent Audoly, Samuel Zwillich, Paul Changelian

**Affiliations:** 1 Pfizer Global Research and Development, MS#8220-2235, Groton, CT 06340, USA; 2 Pfizer Global Research and Development, 50 Pequot Ave, New London, CT 06320, USA; 3 Merck Research Laboratories, West Point, PA 19486, USA; 4 1009 Glenhill Drive, Northville, MI 48167, USA

## Abstract

**Introduction:**

CP-690550 is a small molecule inhibitor of Janus kinase 3 (JAK3), a critical enzyme in the signaling pathway of multiple cytokines (interleukin (IL)-2, -7, -15 and -21) that are important in various T cell functions including development, activation and homeostasis. The purpose of this study was to evaluate CP-690550 in murine collagen-induced (CIA) and rat adjuvant-induced (AA) models of rheumatoid arthritis (RA).

**Methods:**

CIA and AA were induced using standard protocols and animals received the JAK3 inhibitor via osmotic mini-pump infusion at doses ranging from 1.5–15 mg/kg/day following disease induction. Arthritis was assessed by clinical scores in the CIA models and paw swelling monitored using a plethysmometer in the AA model until study conclusion, at which time animals were killed and evaluated histologically.

**Results:**

CP-690550 dose-dependently decreased endpoints of disease in both RA models with greater than 90% reduction observed at the highest administered dose. An approximate ED_50 _of approximately 1.5 mg/kg/day was determined for the compound based upon disease endpoints in both RA models examined and corresponds to CP-690550 serum levels of 5.8 ng/ml in mice (day 28) and 24 ng/ml in rats (day 24). The compound also reduced inflammatory cell influx and joint damage as measured histologically. Animals receiving a CP-690550 dose of 15 mg/k/d showed no histological evidence of disease.

**Conclusion:**

The efficacy observed with CP-690550 in CIA and AA suggests JAK3 inhibition may represent a novel therapeutic target for the treatment of RA.

## Introduction

Rheumatoid arthritis (RA) is a chronic, systemic disease characterized by persistent inflammatory synovitis that typically involves peripheral joints in a symmetric distribution [1]. The synovial inflammation can cause cartilage destruction and bone erosions that are irreversible. To minimize the radiographic damage, it has been recognized that initiation of therapy with disease-modifying antirheumatic drugs (DMARDs) within 3 months after disease diagnosis is critical [2]. The folic acid antagonist methotrexate (MTX) is the DMARD most commonly selected for initial therapy [2] and whose mechanism of action has been attributed, at least in part, to its ability to function as an antimetabolite. As such, the compound inhibits cell proliferation in the inflamed synovium but can affect other proliferating tissues, including gut and bone marrow, producing associated side effects. The use of biological response modifiers, such as tumor necrosis factor (TNF) antagonists, has grown due to efficacy observed in many patients and reasonable safety profile [3]. However, the incomplete efficacy and/or toxicities observed with agents such as these create a need for additional therapies with novel mechanisms of action.

The key role that T cells appear to play in the pathogenesis of the disease has supported evaluation of calcineurin inhibitors such as cyclosporin A and tacrolimus in RA patients [4]. Clinical efficacy for both calcineurin inhibitors has been reported, particularly in combination with other DMARDs such as methotrexate. However, the use of cyclosporine and tacrolimus in this patient population may be limited based upon the multiplicity and severity of associated adverse reactions. CP-690550 is a novel immunosuppressant that has not exhibited the safety liabilities associated with calcineurin inhibition, yet has demonstrated efficacy in a number of animal models including delayed-type hypersensitivity and cardiac allograft rejection [5,6]. CP-690550 is a small molecule inhibitor of the tyrosine kinase Janus kinase 3 (JAK3), an enzyme that is associated with the common gamma chain (γc) of various cytokine receptors and is critical for signal transduction by interleukin (IL)-2, -7, -15 and -21 [7]. Interestingly, JAK3 expression has been shown to decrease in the synovial tissue biopsies from active rheumatoid arthritics receiving and responding to DMARD therapy [8].

Since multiple cytokines whose receptors signal through pathways involving JAK3 have been associated with progression of arthritis, experiments were designed to evaluate the effects of CP-690550 in rodent models of the disease. Neither murine collagen-induced arthritis (CIA) nor adjuvant-induced arthritis (AA) in rats are identical to RA, but both share the common features of inflammation of the synovial membrane, erosion of bone, and cartilage degradation. In both models of RA, we observed dose-dependent inhibition of disease endpoints that correlated with reduction in histological changes. These data support JAK3 inhibition as a new target for the treatment of RA.

## Materials and methods

### Reagents

CP-690550 was synthesized in-house and the enzyme specificity of this compound has been previously described [5]. The anti-TNF antibody TN.1912 has been shown to effectively neutralize TNF *in vivo *and to have a 7-day half-life [9]. This clone was scaled up in-house and the dose of agent chosen for this study based upon internal (data not shown) and external experiments demonstrating efficacy in the CIA model at doses ranging from 300 μg/mouse intraperitoneally once a week to 300 μg/mouse intraperitoneally twice a week [10-12]. Unless otherwise specified, reagents were purchased from Sigma-Aldrich Chemical Company (St. Louis, MO, USA).

### General animal care

For collagen-induced arthritis studies, male DBA/J1 mice (7–9 weeks old from Jackson Labs, Bar Harbor, ME, USA) were used. For studies of adjuvant-induced arthritis, male Lewis rats were used (~50–60 days old from Charles River Labs, Wilmington, MA, USA). Animals were housed in standard cages with access to food and water *ad libitum*. The environment was maintained at 21 ± 2°C with a time regulated light period from 6 am to 6 pm. Studies were conducted in accordance with the guidelines set forth by the Pfizer Animal Care and Use Committee. An additional CIA study using mice of same age, strain and source was performed at Boulder BioPATH Inc as described below.

### Murine CIA experiment

Male DBA/J1 mice were shaved at the base of the tail and injected with 0.1 ml emulsion consisting of a 1 to 1 (1 mg/1 mg) mixture of type II chicken collagen with *Mycobacterium butyricum *(Difco lot # 147539, Voigt Global Distribution, Lawrence, Kansas) as an adjuvant. Three weeks later, the mice were boosted with another 0.1 ml injection of emulsion at the base of the tail to induce disease. Three days following this injection, the animals were randomized and Alzet osmotic mini-pumps (28-day pumps, model 2004, Durect Corporation, Cupertino, CA) were implanted subcutaneously on the back of each mouse to deliver CP-690550 at 1.5 (*n *= 13), 5 (*n *= 14) or 15 (*n *= 14) mg/kg/day, poly(ethylene glycol) (PEG)300 vehicle (*n *= 15) or no pump (*n *= 11). It was necessary to administer CP-690550 via osmotic mini-pumps due to the poor pharmacokinetic (PK) properties of this compound in rodents. The mice were scored in a blinded manner (0–12) twice weekly for 3 weeks for signs of arthritis in each paw according to the following scale: 0 = no swelling or redness/normal paw; 1 = swelling and/or redness in one digit; 2 = swelling and/or redness in two or more digits; and 3 = entire paw is swollen or red. Upon study completion (day 28), mice were killed with CO_2_. Blood samples were immediately taken via cardiac puncture and serum analyzed for CP-690550 levels. Following this, the knees were removed and processed for histological analyses as described below. The knees were chosen instead of the paws because both our lab and others [13] have observed a good correlation between paw swelling and histological changes.

### Boulder BioPATH CIA experiment

An additional CIA study was performed at Boulder BioPATH (Boulder, CO, USA) as described above with the following modifications: (a) inclusion of anti-TNF treatment group (250 μg/animal intraperitoneally twice a week); (b) collection of interim serum samples on day 15; (c) increase in study length from 28 (Pfizer study) to 31 days; and (d) mice were scored in a blinded manner on a 0–20 scale twice weekly for 3 weeks for signs of arthritis in each paw (*n *= 10 for all groups except naïve where *n *= 5). Clinical signs were evaluated using the following scale: 0 = normal; 1 = one joint affected or mild diffuse erythema and swelling; 2 = two joints affected or mild diffuse erythema and swelling; 3 = three joints affected or mild diffuse erythema and swelling; 4 = four joint affected or marked diffuse erythema and swelling; and 5 = severe erythema and severe swelling.

### Rat AA

Male Lewis rats were shaved at the base of the tail and injected once intradermally with 100 μl of a 10 mg/ml *Mycobacterium butyricum *(Difco lot # 147539) mineral oil suspension. Ten days after this injection, the foot volumes of both the right and left paws were measured with a Stoelting plethysmometer and Alzet osmotic mini-pumps (14-day pumps, model 2ML2 (Stoeling Company, Wood Dale, IL) were implanted subcutaneously to deliver CP-690550 1.5, 5 or 15 mg/kg/day or vehicle (PEG300) (*n *= 10 for all groups except naïve where *n *= 5). Swelling in the paws of the rats was measured in a blinded manner with a plethysmometer twice weekly for 2 weeks. At the completion of the study (day 24), rats were killed with anesthesia. Blood samples were immediately taken via cardiac puncture and serum analyzed for CP-690550 levels. Following this, the hind paws were removed and processed for histological analyses as described below.

### Histology

Mouse hind limbs and rat hind paws were collected and immersion fixed in 10% buffered formalin. Limbs and paws were routinely processed, embedded in paraffin, sectioned and analyzed as previously described [14].

### IL-6 analysis

Serum IL-6 levels were measured by enzyme-linked immunosorbent assay (ELISA) using a murine IL-6 kit (Quantikine; R&D Systems, Minneapolis, MN, USA). The number of animals available for IL-6 measurements was as follows: naïve (*n *= 3); vehicle (*n *= 8); anti-TNF (*n *= 8); CP-690550 1.5 (*n *= 6), 5 (*n *= 8) or 15 (*n *= 7) mg/kg/day.

### Drug level analysis

Serum concentrations of CP-690550 were determined using reverse-phase high performace liquid chromatography (HPLC) with MS/MS (mass spectrometry/mass spectrometry) detection as previously described [5]. Since CP-690550 was administered via osmotic mini-pumps, the terminal drug concentration represents the steady-state drug concentrations in these animals.

### Statistical analysis

Scores for all measurements were analyzed by one sample t test (Statview v.5, SAS Institute, Cary, NC, USA) and significance set at p ≤ 0.05.

## Results

### Murine CIA

#### Clinical signs

**Figure 1:**
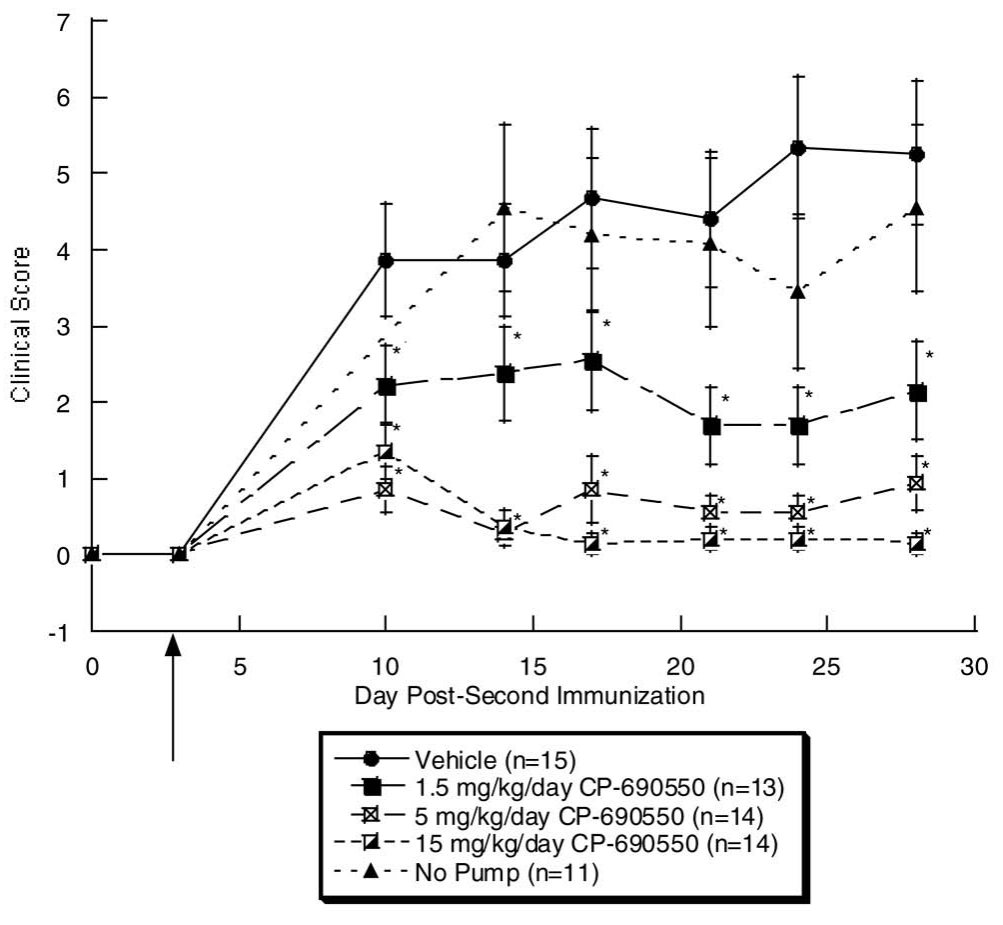
Clinical scores from murine collagen-induced arthritis study 1. Animals were given initial injection of type II collagen on day -21 and disease was induced with a second injection on day 0. On day 3 (arrow), pumps were implanted and clinical signs measured twice a week from day 10 to day 28. By day 10, a statistically significant, dose-dependent decrease in the clinical score was observed with all doses of CP-690550 and these remained significant throughout the remainder of the study.

In the first murine CIA study, an increase in clinical signs of disease were detected on day 10. The vehicle treated mice attained a clinical score of 3.9 ± 0.7 that gradually increased to a maximum of 5.3 ± 0.9 on day 27 (Figure 1). Clinical scores were similar in diseased animals not receiving a pump, suggesting neither implantation of the pump nor the vehicle had a significant effect on the clinical score. At the lowest dose of CP-690550 (1.5 mg/kg/day), the clinical score peaked on day 10 at 2.2 ± 0.5 and the response remained attenuated relative to the control group for the remainder of the study. Treatment at both the intermediate (5 mg/kg/day) and high (15 mg/kg/day) doses of CP-690550 produced a highly significant, near total suppression of clinical scores throughout the entire study. Based upon the clinical scores, the ED_50 _of CP-690550 was ~1.5 mg/kg/day with > 90% disease reduction observed at the 15 mg/kg/day dose.

**Figure 2:**
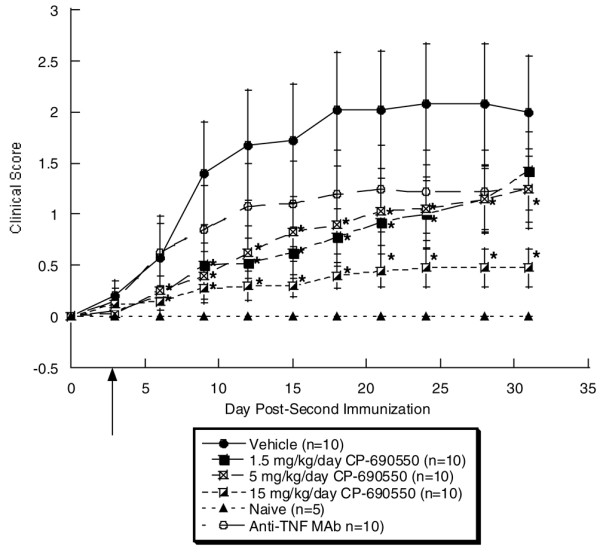
Clinical scores from murine collagen-induced arthritis study 2. Animals were given initial injection of type II collagen on day -21 and disease was induced with a second injection on day 0. On day 3 (arrow), pumps were implanted and clinical signs measured twice a week from day 6 to day 31. By day 9, a statistically significant, dose-dependent decrease in the clinical score was observed with all doses of CP-690550 and these remained significant to day 28. On day 31, only the high and mid dose of CP-690550 contained a statistically significant decrease in clinical score. At no time point during the course of the study did the anti-tumor necrosis factor (TNF) antibody treatment result in a statistically significant decrease in clinical score.

A second murine CIA study was performed and included an anti-TNF treatment group as a comparator. The clinical scores were reduced in this study relative to the first CIA study, which could be due to subjective differences in scoring. As early as 3 days post-implantation of pumps, mice receiving both high and low doses of CP-690550 exhibited significant reductions in the clinical score vs vehicle (Figure 2). By days 9–28 all three dose levels of CP-690550 resulted in a significant reduction in the clinical score. On day 31, only the high and mid-dose of CP-690550 maintained this statistically significant reduction in clinical score vs vehicle. Although there was a trend, at no time point in the study did treatment with anti-TNF (250 μg/mouse) result in a statistically significant decrease in the clinical score over vehicle.

#### Histological changes

**Figure 3:**
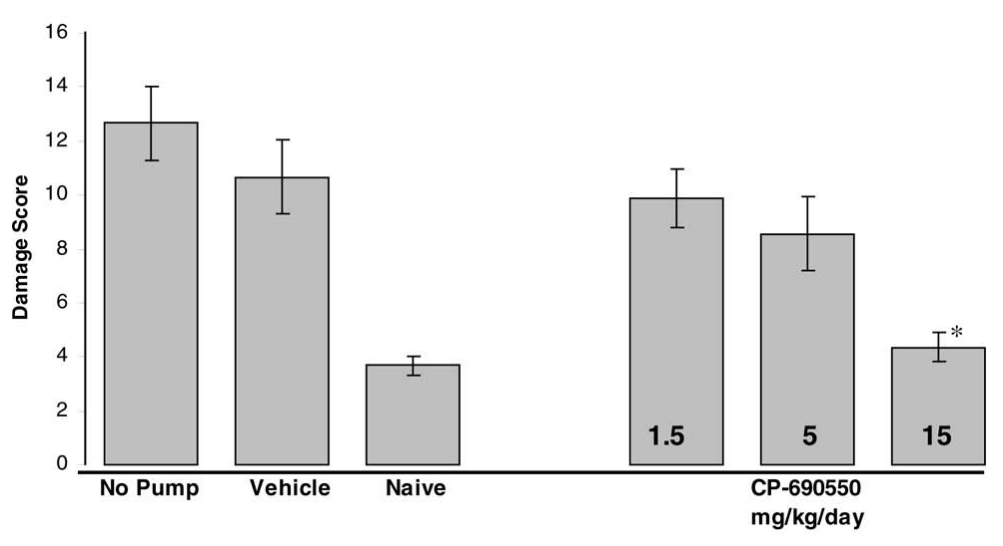
Histological evaluation of damage to murine knees. Histological sections of knee samples from murine collagen-induced arthritis study 1 were graded as described in Table 1. CP-690550 produced a dose-dependent inhibition of knee damage that reached statistical significance (p < 0.0001) at the 15 mg/kg/day dose relative to vehicle.

**Figure 4:**
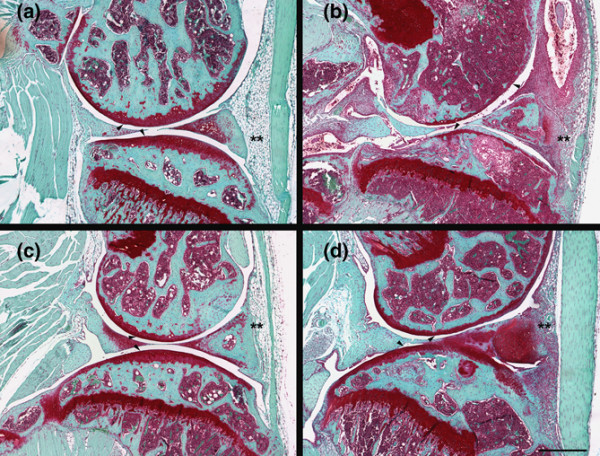
Representative histological sections from murine collagen-induced arthritis study 1. In all figures, the arrowheads point to the femoral condyle (top) and tibial plateau (bottom) articular cartilages and the asterisks highlight the inflammatory cells in the soft tissue surrounding the joint. Panel **(a)** is from a naïve control containing no damage to the articular cartilage and few cells in the soft tissue surrounding the joint. Panel **(b)** is from a vehicle treated animal, demonstrating significant influx of inflammatory cells into the synovial tissue and cavity as well as significant proteoglycan loss and erosion of the articular cartilage. Panels **(c)** and **(d)** are from animals that have been dosed with CP-690550 at 15 and 1.5 mg/kg/day, respectively. At both dose levels, CP-690550 decreased cell influx, synovial hypertrophy, articular cartilage damage and proteoglycan loss. The knees from animals dosed with CP-690550 15 mg/kg/day were very similar to the knees from the naïve animals. Bar = 500 μm.

In the first CIA study, inflammation and damage to the knee joint were assessed histologically on blinded sections and joint damage scores (0–15) assigned based upon the scoring key in Table 1. The knees from naïve control animals were unremarkable and had a mean damage score of 3.7 ± 0.3 (Figure 3). In contrast, in both no pump (12.7 ± 1.4) and PEG 300 vehicle alone (10.7 ± 1.4) treatment groups, portions of the non-calcified cartilage had been worn down to the tidemark and significant cell influx and synovial hypertrophy were observed. In regions where the non-calcified articular cartilage was still present, it was extensively depleted of proteoglycan and devoid of chondrocytes. Treatment with CP-690550 resulted in a dose dependent reduction in the inflammation and damage to the articular cartilage (Figure 4). The average histological damage scores in the CP-690550 treated mice ranged from 9.8 at 1.5 mg/kg/day to 4.4 at 15 mg/kg/day (Figure 3). The histologically determined ED_50 _dose of CP-690550 was approximately 6.5 mg/kg/day.

**Table 1:** Scoring key for murine knees

**Score**	**Edge**	**Proteoglycans**	**Open lacuna**	**Non-calcified layer**	**Synovial lining**	**Blood in cavity**	**Pannus tongues**
0	Smooth	> 90% present	None	> 90% present	1 even	Absent	Absent
1	Rough	> 50% present	Some	> 50% present	< 50% 2 layers	Present	Present
2		< 50% present	Many	< 50% present	> 50% 2 layers		
3		< 10% present		< 10% present	2 layers		
4					> 2 layers		

In the second CIA study, the clinical score data correlated with the histological results from the four paws in that the greatest efficacy was observed with the 15 mg/g dose of CP-690550 (84% inhibition) while the mid and low doses of CP-690550 were statistically equivalent to treatment with anti-TNF (45 % inhibition).

#### Serum IL-6 levels

**Figure 5:**
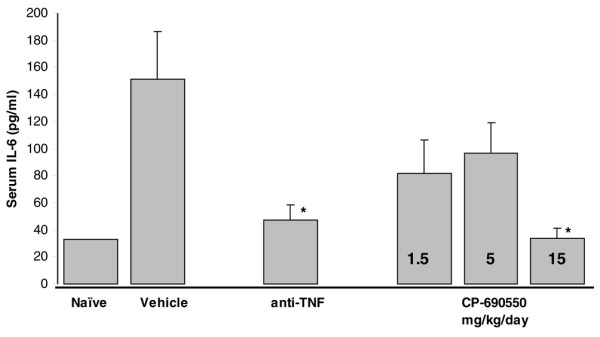
Serum interleukin (IL)-6 levels from murine collagen-induced arthritis study 2. Blood was drawn from mice 15 days following the second type II collagen injection and serum IL-6 measured by enzyme-linked immunosorbent assay (ELISA). Data are mean ± standard error of the mean of values from 6–8 animals/treatment group, except the naïve group (*n *= 3).

Serum IL-6 levels were measured in the second CIA study and were found to be elevated ~4.6-fold in diseased control mice vs naïve mice (Figure 5). Whereas lower doses of CP-690550 trended towards a reduction in IL-6 levels, only the 15 mg/kg/day group produced a statistically significant effect. Administration of the anti-TNF was also significantly effective at lowering serum IL-6 levels.

### Rat AA

#### Clinical changes

**Figure 6:**
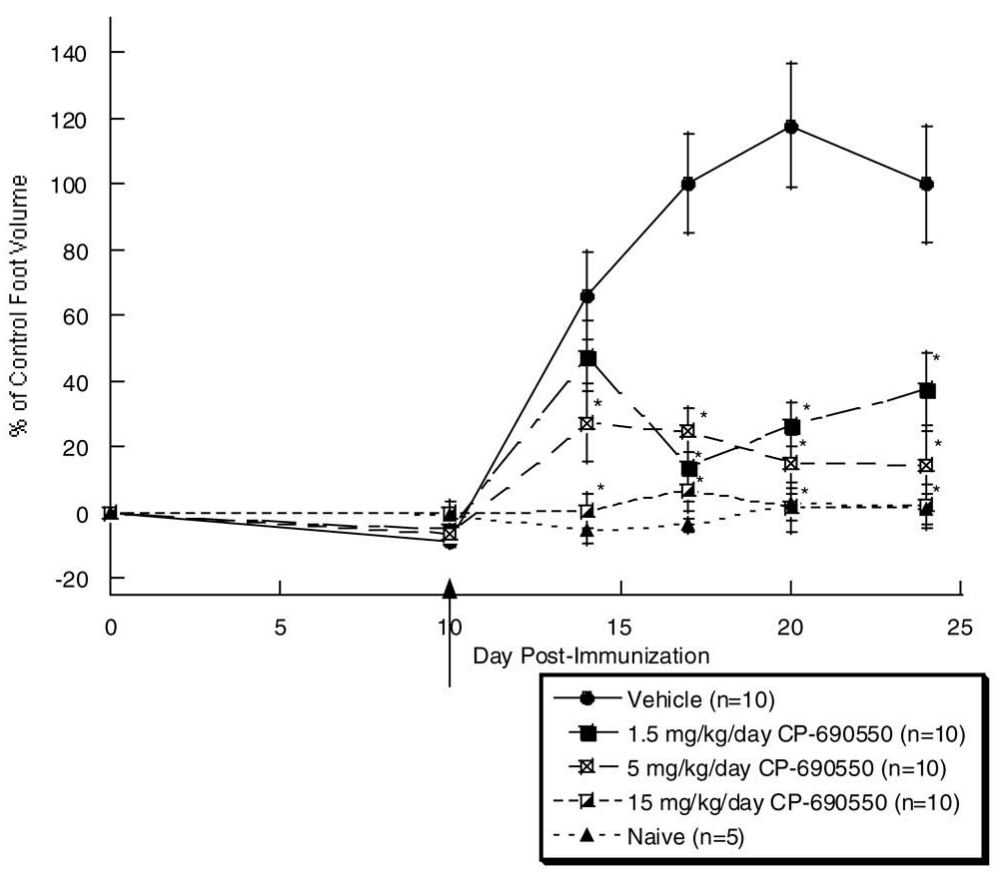
Measurement of rat foot pad swelling in rat adjuvant-induced arthritis (AA) model. Animals were injected at the base of the tail with *Mycobacterium butyricum *in mineral oil on day 0 to induce disease. On day 10 (arrow), pumps were implanted and foot swelling measured twice a week from day 14 to day 24. CP-690550 decreased hind paw swelling in a dose dependent manner. In the rat AA model, data were expressed as percent control with the diseased vehicle treatment group on day 24 being set to 100%. As early as day 14, both the 5 and 15 mg/kg dose of CP-690550 resulted in a statistically significant decrease in foot volume. By day 17, all doses of CP-690550 resulted in a statistically significant decrease in foot volume that remained significant for the rest of the study. Data are mean ± standard error of the mean of 10 animals per group, except for the naïve group (*n *= 5).

By day 14 after adjuvant administration in the rat AA model, paw swelling was evident in all rats except those receiving CP-690550 at 15 mg/kg/day. Treatment with CP-690550 produced a dose-dependent inhibition of footpad swelling (Figure 6). Near complete inhibition was achieved at both the 5 and 15 mg/kg dose levels at all time points. Swelling in the 1.5 mg/kg dose level was reduced relative to vehicle from days 7–14.

#### Histological changes

**Figure 7:**
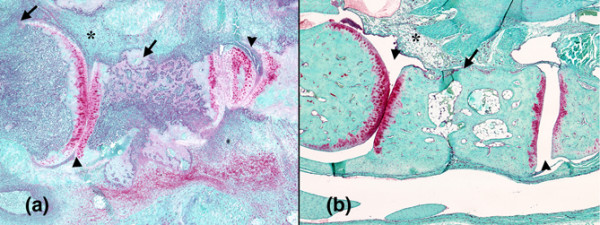
Histological evaluation of damage to foot pad in rat adjuvant-induced arthritis (AA) model. Representative sections from vehicle **(a)** and 15 mg/kg/day CP-690550 **(b)** treated rats on day 24. In the vehicle treated animal there was significant inflammation (asterisk) and destruction of the metatarsal bone (arrow) and joint spaces (arrowhead). In contrast, there was no inflammation and the metatarsal joint spaces appeared normal in the CP-690550 treated animal.

Histological evaluation of the hind paws revealed significant inflammation and damage present in the vehicle dosed animals (Figure 7). The bones and joint cavities from the first metatarsal to the tibia on the medial side of the foot were evaluated on a 0–8 scale using a modified scoring key (Table 2). Only the feet from the vehicle and CP-690550 15 mg/kg/day animals were evaluated histologically. A significant reduction was observed in the damage score in the CP-690550 15 mg/kg/day treated group (2.4 ± 0.3 damage score) vs the vehicle treated group (5.9 ± 0.6 damage score).

**Table 2:** Modified scoring key for rat foot pad damage

**Score**	**Proteoglycan retention**	**Inflammation**	**Bone damage**
0	> 90% present	Few cells	Normal
1	> 50% present	Slight to moderate	Questionable damage
2	Only chondrocytes stain	Moderate to heavy	Damaged
3	< 10% present	Extremely heavy	

#### Drug levels in serum

In the first murine CIA study, serum levels of CP-690550 on day 28 ranged from 6 ng/ml at 1.5 mg/kg/day to 70 ng/ml at 15 mg/kg/day (Table 3). In the second CIA study, equivalent doses of CP-690550 produced approximately 50% less drug in the serum on day 31. In the rat, equivalent doses of CP-690550 produced greater than fourfold higher drug levels than in the mouse (Table 3).

**Table 3:** CP-690550 serum levels (ng/ml)

**Dose (mg/kg/d)**	**Mouse study 1**^a^	**Mouse study 2**^b^	**Rat study**
15	70 ± 17	37 ± 5	259 ± 41
5	27 ± 6	17 ± 2	124 ± 16
1.5	6 ± 1	< LLOQ	24 ± 5

Data are mean ± standard error of the mean. All samples were collected at the termination of the study. *n *= 10 for all groups. LLOQ, lower limit of quantification.

^a^LLOQ = 5 ng/ml (Pfizer study).

^b^LLOQ = 10 ng/ml (Boulder BioPath study).

## Discussion

CP-690550 produced significant dose-dependent attenuation of inflammatory swelling, cell influx and cartilage damage in two well characterized rodent models. A T cell contribution to disease has been demonstrated in both models [15-17]. In murine CIA, the magnitude of effects observed at the highest dose of the JAK3 inhibitor tested (15 mg/kg/d) were greater than those following administration of anti-TNF antibody (TN-1912) when assessing clinical scores and histology. The magnitude of effect of anti-TNF that we observed on the clinical arthritis score is consistent with that reported previously [10-12] when animals were dosed with the same anti-TNF mAb. Anti-TNF treatment is efficacious in murine CIA when dosed before or immediately after the onset of CIA (see review of the role of TNF and IL-1 in CIA; [18]). Even though we did begin treating the mice immediately after disease induction, the fact that anti-TNF treatment was not as efficacious as treatment with CP-690550 in murine CIA could be due to the role of IL-1 or other inflammatory mediators in this animal model.

CP-690550 doses/exposures that produced effects in this model are consistent with those demonstrating immune suppression in other murine models including delayed-type hypersensitivity and cardiac allograft transplantation [5,6]. Interestingly, both CP-690550 (78% reduction vs control) and the anti-TNF mAb (68% reduction vs control) significantly reduced serum IL-6 levels. IL-6 has been proposed to play an important role in the development of CIA based upon delay in onset and reduction in disease magnitude observed in mice genetically deficient in this cytokine [19]. The effects of anti-TNF on IL-6 are consistent with other reports in which inhibition of TNF action, either via genetic ablation of its receptor [20] or via anti-TNF mAb [21,22] were found to down-modulate levels of IL-6. However, in our studies, anti-TNF mAb treatment reduced serum IL-6 by a similar magnitude as CP-690550 but did not demonstrate the same degree of efficacy, which suggests the JAK3 inhibitor, affected other inflammatory mediators important for expression of disease in this model. A role for IL-6 in rheumatoid arthritis has been proposed based upon the ability of the cytokine to activate inflammatory responses and osteoclastogenesis and is supported by positive clinical data obtained with the anti-IL-6 mAb tocilizumab in this patient population [23].

The efficacy produced by CP-690550 in the rodent models of arthritis may result from its ability to affect signaling of a number of cytokines including IL-2, -7, -15 and -21 as a consequence of JAK3 inhibition [5]. IL-2 mRNA was found to be markedly increased in arthritic paws from mice with CIA during the early phases of disease [24]. This may explain the efficacy observed following prophylactic administration of an anti-IL2R antibody in this model [25]. When mice with established disease were treated with cyclosporine 50 or 75 mg/kg/day, disease was also attenuated [26]. Tacrolimus is another, albeit more potent, calcineurin inhibitor that has also demonstrated efficacy in experimental models of rheumatoid arthritis [27]. In rat arthritis models, tacrolimus suppressed paw inflammation, type II collagen antibody formation and delayed-type hypersensitivity to type II collagen [27,28]. While clinical trials of tacrolimus in rheumatoid arthritis have been conducted, it appears that the compound has a narrow therapeutic window which limits its utility [29].

IL-15 is a cytokine with close homology to IL-2 whose receptor shares signaling through the common gamma chain. Previous studies from our lab have demonstrated that CP-690550 inhibits IL-15-mediated up-regulation of activation markers on CD8+ T cells and NK cells [30]. Upon chronic treatment with CP-690550, there is a preferential loss of these cells from the circulation, which is consistent with a role for IL-15 in their survival [6,30]. Evidence is emerging for the importance of IL-15 in the pathogenesis of rheumatoid arthritis. Elevated serum levels of the cytokine have been reported in arthritic patients, the primary source of which may be macrophages residing in the synovial lining layer of inflamed joints [31]. IL-15 produces a number of effects which may be relevant to the pathogenesis of arthritis including recruitment and activation of T lymphocytes into the synovial membrane and induction of TNFα production [32,33]. A soluble fragment of the murine IL-15Rα chain inhibited development of collagen-induced arthritis in DBA/1 mice [34]. Administration of an IL-15 mutant/Fcγ2c fusion protein in established murine CIA blocked disease progression and reduced long term articular inflammation and destruction [33]. The therapeutic benefit achieved by inhibiting IL-15 is supported by evidence that HuMax-IL-15, a fully human anti-IL-15 mAb, produced encouraging signs of efficacy in rheumatoid arthritis patients [35].

IL-21 is a cytokine produced by activated CD4+ T cells that also signals through JAK3. It enhances T cell activation, proliferation and secretion of pro-inflammatory cytokines such as TNFα and IL-21R has been shown to be over-expressed in inflamed synovial membrane and peripheral blood or synovial fluid leukocytes of rheumatoid arthritis patients [36]. A recent publication reported that blockade of IL-21 effects with a murine IL-21 receptor Fc fusion protein attenuated disease in both mouse and rat models of arthritis [22]. Effects in a 'semi-therapeutic' murine CIA model (compound administration begun when 10% of mice began to exhibit clinical signs of disease) included reduction in disease severity scores (including histology) and serum IL-6 levels. Effects produced by IL-21RFc were even more profound in a rat adjuvant-induced arthritis model in which full amelioration of clinical signs was achieved in conjunction with significant reduction in histological damage [22]. Recent evidence demonstrates that IL-21 is a key cytokine involved in the generation of Th17 cells which have been shown to mediate tissue inflammation via production of IL-17 [37,38]. Thus it is possible that CP-690550, through inhibition of IL-21R signaling, may also be efficacious in the CIA model by reducing IL-17 producing Th17 cells which have been proposed to play an important role in the pathogenesis of autoimmune diseases.

IL-7 represents another member of the IL-2 family that signals through the common gamma chain. It plays a key role in T cell homeostasis supporting growth, proliferation and survival of developing and mature T cells. In mice, unlike humans, its absence or blockade results in a diminution of B cell numbers as was evident in our own studies that examined the effects of chronic CP-690550 administration on circulating lymphocytes [6]. IL-7 has also been suggested to play a role in rheumatoid arthritis based upon the observation of increased levels of the cytokine in this patient population, its ability to induce TNFα and induction of bone loss by stimulation of RANKL-dependent osteoclastogenesis [39].

The potential for CP-690550 to attenuate multiple cytokines associated with rheumatoid arthritis by virtue of its ability to inhibit JAK3 may provide improved efficacy vs a single agent. For example, TNF antagonists rarely induce complete disease remission and not all patients respond to TNF-blocking therapies [3]. IL-1 antagonism also demonstrates some effectiveness albeit to a lesser extent than TNF blockers [40]. However, combined inhibition of these two cytokines has been shown to provide increased benefit relative to inhibition of either alone [41]. Hence, JAK3 inhibition provides a potentially beneficial target for the treatment of RA based upon its ability to inhibit multiple cytokines known to be involved in the pathogenesis of the disease.

## Conclusion

CP-690550, a potent inhibitor of JAK3, reduced the clinical and histological manifestations of joint inflammation, including bone and cartilage damage, when administered therapeutically in murine CIA and rat AA. The effects of CP-690550 were dose dependent and higher doses were required for suppression of CIA histopathology than clinical manifestations. These data support the evaluation of CP-690550 for DMARD activity in RA patients.

## Abbreviations

AA = adjuvant-induced arthritis; CIA = collagen-induced arthritis; DMARD = disease-modifying antirheumatic drug; ELISA = enzyme-linked immunosorbent assay; IL = interleukin; JAK3 = Janus kinase 3; LLOQ = lower limit of quantification; RA = rheumatoid arthritis; TNF = tumor necrosis factor.

## Competing interests

All authors were, or currently are employed by Pfizer Global Research and Development. Pfizer is financing the publication of this manuscript.

## Authors' contributions

AJM was responsible for histology, data analysis and manuscript writing, EK for data analysis and manuscript writing, LA for all Pfizer *in vivo *animal work, SZ for assistance with manuscript writing, and PC for concept and assistance with manuscript writing.
